# Dynamics to Equilibrium in Network Games: Individual Behavior and Global Response

**DOI:** 10.1371/journal.pone.0120343

**Published:** 2015-03-24

**Authors:** Giulio Cimini, Claudio Castellano, Angel Sánchez

**Affiliations:** 1 Grupo Interdisciplinar de Sistemas Complejos (GISC), Departamento de Matemáticas, Universidad Carlos III de Madrid, Leganés, Spain; 2 Istituto dei Sistemi Complessi (ISC-CNR) UoS “Sapienza” Università di Roma, Italy, Rome; 3 Instituto de Biocomputación y Física de Sistemas Complejos (BIFI), Universidad de Zaragoza, Zaragoza, Spain; University of Maribor, SLOVENIA

## Abstract

Various social contexts can be depicted as games of strategic interactions on networks, where an individual’s welfare depends on both her and her partners’ actions. Whereas much attention has been devoted to Bayes-Nash equilibria in such games, here we look at strategic interactions from an evolutionary perspective. To this end, we present the results of a numerical simulations program for these games, which allows us to find out whether Nash equilibria are accessible by adaptation of player strategies, and in general to identify the attractors of the evolution. Simulations allow us to go beyond a global characterization of the cooperativeness at equilibrium and probe into individual behavior. We find that when players imitate each other, evolution does not reach Nash equilibria and, worse, leads to very unfavorable states in terms of welfare. On the contrary, when players update their behavior rationally, they self-organize into a rich variety of Nash equilibria, where individual behavior and payoffs are shaped by the nature of the game, the social network’s structure and the players’ position within the network. Our results allow to assess the validity of mean-field approaches we use to describe the dynamics of these games. Interestingly, our dynamically-found equilibria generally do not coincide with (but show qualitatively the same features of) those resulting from theoretical predictions in the context of one-shot games under incomplete information.

## Introduction

Strategic interactions between people encompass several social and economic contexts (ranging from public goods provision to information collection) where an individual’s welfare depends both on her own actions and on the actions taken by her interacting partners. Much effort has been devoted to understand how the pattern of social connections shapes the choices that individuals make and the payoffs that they can earn [1–7] (see [8–10] for an overview of the field). On the theoretical side, the traditional approach of identifying the Bayes-Nash equilibria in one-shot network games is problematic due to the existence of multiple equilibria (even for very small network sizes), which imply a huge range of possible outcomes. Nevertheless, it has been shown that the problem of multiplicity can be resolved sometimes by the introduction of incomplete information [11, 12]—which in the context of network games means having only a local knowledge of the network: a player is aware of the number of her connections (her degree) but not of the degrees of the others. The key point is that when players have limited information about the network they are unable to condition their behavior on its fine details, and this leads to a significant simplification and sharpening of equilibrium predictions. A general framework for the study of multiplayer games on networks under incomplete information has been recently developed in [13, 14]. In these works, the authors consider two canonical types of interaction: the best-shot game and the coordination game, as representatives for strategic substitutes and strategic complements, respectively [15]. These two cases represent alternative scenarios on how a player’s payoff is affected by the actions of others, covering many of the game-theoretic applications studied in the economic literature. For both these games in their one-shot version (*i.e.*, for which equilibrium strategies are played once and for all), a context in which players have complete information on the social network allows for a very rich set of Nash equilibria, where the relation between network connections, equilibrium actions and payoffs may exhibit very different patterns [13, 14]. In contrast, by relaxing the assumption of complete information, Galeotti et al. [14] proved the existence of Bayes-Nash equilibria involving strategies that are monotonic with respect to players’ degrees and symmetric, *i.e.*, with all players of the same degree *k* choosing the same strategy.

In this work, instead of focusing on equilibrium strategies played once and for all, we look for the Nash equilibria of these games that are dynamically accessible, *i.e.*, we consider iterated network games within an evolutionary framework [16] (see [17–19] for an overview of the field): players’ own actions are described by a strategy which is subject to an evolutionary process [20, 21], meaning that—at odds with one-shot games—players’ actions may change at each repetition of the game. It is important to note that this is different from the stochastic stability approach presented for this type of games in [22], where the focus is on the different time regimes of the evolution. In fact, our framework represents a way to select among the large multiplicity of equilibria that exist in network games in a spirit very close to that of biological evolution. In particular, we consider two main mechanisms for players to adapt their strategy: imitation and rational deduction, represented respectively by Proportional Imitation (PI) [23] and Best Response (BR) [24, 25] dynamics. In both cases, agents play multiple instances of the game, and can adapt their strategy hoping to increase their payoffs. Note, however, that agents do not make strategic considerations about the network’s global structure and their position within it; instead, they base their decision on their own actions/payoffs and on those of neighbors, as observed in the past.

Under the above framework, our main contribution is the study of the resulting evolutionary dynamics of these games as well as their stationary configurations. In particular, we are able to understand which Nash equilibria are dynamically accessible by adaptation of players’ strategies, and in general to identify the strategy configurations that are the attractors of the dynamics—either Nash equilibria or, possibly, stationary states of different nature. We achieve these results by running a numerical simulation program that is intended to represent the evolutionary system. As a reference, we consider the analytical results we obtained in [26], where we studied this issue by resorting to two mean field approaches, the usual homogeneous one (MF), and its heterogeneous version (HMF) [27]. As shown below, the numerical simulations allow us to assess the accuracy of the pictures provided by the two approaches and, furthermore, to gain more insight on the individual behavior of the different types of players—going far beyond the information that can be obtained from analytical predictions. In a nutshell we find that, for the best-shot game, imitation leads necessarily to full defection—which is however not a Nash equilibrium—whatever the underlying network, whereas, rational deduction allows players to self-organize into a variety of Nash equilibria, with heterogeneous networks enhancing the total amount of cooperation. On the other hand, the behavior for the coordination game depends mainly on how much players reinforce each other, which generally results in a phase transition between a fully defective Nash equilibrium and full cooperation (which is not always Nash). Notably, the transition point goes to zero for increasing network heterogeneity also when the incentive to cooperate vanishes.

In addition to these results, we compare our approach with the theoretical framework of [14] for one-shot games under incomplete information. We show that the Nash equilibria predicted by their theory generally do not coincide with the ones obtained by evolutionary dynamics; nevertheless, these equilibria possess qualitatively the same features of those resulting from evolution, in terms of players’ strategies with respect to their neighborhood structure.

## Methods

### Network Games

We start by formalizing the games we use to model strategic interactions. Consider a society of *n* agents, placed on the nodes of a social network. The links between agents reflect social interactions, and connected agents are said to be *neighbors*. Each individual must choose (independently) an action in *X* = {0, 1}, where action 1 may be interpreted as *cooperating* and action 0 as not doing so—or *defecting*. To define the payoffs, let *x*_*i*_ be the action chosen by agent *i*, *N*_*i*_ the set of *i*’s neighbors, *x*_*N*_*i*__ = ∑_*j* ∈ *N*_*i*__ *x*_*j*_ the aggregate action in *N*_*i*_, and *y*_*i*_ = *x*_*i*_+*x*_*N*_*i*__. There is a cost *c*, where 0 < *c* < 1, for choosing action 1, while action 0 bears no cost. For strategic substitutes, the payoff function takes the form:

$$\begin{array}{c} \pi_{i}=H\left(y_{i}-1\right)-c\;x_{i}, \end{array}$$ (1)

where *H*(⋅) is the Heaviside step function *H*(*x*) = 1 if *x* ≥ 0 and *H*(*x*) = 0 otherwise. Strategic substitutes thus represent an anti-coordination game: a player would prefer that someone of her neighbors takes action 1 (rather than taking the action herself), but she would be willing to take action 1 if nobody in the neighborhood does. In this way, strategic substitutes encompass many scenarios that allow for free riding or have a public good structure of play. Instead, strategic complements arise whenever the benefit that an individual obtains from undertaking a given behavior is greater as more of her partners do the same: they represent a coordination game, where the payoff reads:

$$\begin{array}{c} \pi_{i}=\left(\alpha x_{N_{i}}-c\right)\;x_{i}. \end{array}$$ (2)

with 0 < *α* < *c* representing the incentive to chose action 1.

### Bayes-Nash Equilibria

We now recall the framework of Galeotti et al. [14] to describe strategic interactions for one-shot games (*i.e.*, where players choose their action *x* once and for all) under incomplete information. The basic assumption is that each player knows only her own degree *k*′ and the probability distribution *P*(*k*∣*k*′) of the degree *k* of her neighbors—which, for uncorrelated networks (where degrees of neighboring nodes are independent), reads $P(k|k^{\prime })=kP(k)/\bar{k}$ (with $\bar{k}=\sum_{k}kP(k)$ indicating the average connectivity). Within the framework of Bayesian games, a player’s (pure) strategy *σ* ∈ *X* now must depend only on the degree *k* of the player. In fact, for strategic substitutes, if an agent of degree *k* chooses action 1 in equilibrium, it must be because she does not expect that any of her neighbors will choose action 1. Therefore, in an uncorrelated network, an agent of degree *k*−1 faces a lower likelihood of an arbitrary neighbor choosing the action 1, and would be best responding with action 1 as well. In particular, any Nash equilibrium is characterized by a threshold *τ*, the smallest integer for which

$$\begin{array}{c} 1-{\left[1-\sum_{k=1}^{\tau }\frac{kP(k)}{\bar{k}}\right]}^{\tau }\geq 1-c, \end{array}$$ (3)

and an equilibrium *σ* must satisfy *σ*(*k*) = 1 for all *k* < *τ*, *σ*(*k*) = 0 for all *k* > *τ* and *σ*(*τ*) ∈ {0, 1} (*i.e.*, *σ*(*k*) is non-increasing). Instead in the case of strategic complements, independence of neighbor degrees implies that for each player the probability that a random neighbor chooses the action 1 cannot depend on her own degree (the player’s neighbors do not know her degree and, consequently, cannot know whether or not it will be convenient for her to chose action 1). Thus, the expectation of the sum of actions *x*_*N*_*i*__ of any agent *i* with ∣*N*_*i*_∣ = *k* neighbors is increasing in *k*. The structure of payoffs then assures that if a degree *k* agent is choosing the action 1 in equilibrium, any agent of degree greater than *k* must be best responding with the action 1 as well, as she should have as many cooperating neighbors as the agent of degree *k*. Therefore every equilibrium is characterized by an integer threshold *τ* such that

$$\begin{array}{c} \alpha \left(\tau -1\right)\sum_{k=\tau }^{n-1}\frac{kP(k)}{\bar{k}}<c\;\text{and}\;\alpha \tau \sum_{k=\tau }^{n-1}\frac{kP(k)}{\bar{k}}\geq c. \end{array}$$ (4)

and must satisfy *σ*(*k*) = 0 for all *k* < *τ*, *σ*(*k*) = 1 for all *k* > *τ* and *σ*(*τ*) ∈ {0, 1} (in particular, *σ*(*k*) is non-decreasing).

### Evolutionary Dynamics

In this work, instead of considering one-shot games played once and for all, we study iterated games within an evolutionary framework. This means that we consider a supergame consisting in many repetitions (rounds) of the one-shot game, and that at each generic round *t* players may change their strategy (*i.e.*, change their chosen action *x*) according to the behaviors they observed in the past. Here we give the details of the two different mechanisms for strategy updating we consider.

PI is a rule of imitative nature in which each player *i* may assume the strategy *x*_*j*_ of a selected counterpart *j*, which is chosen randomly among the *N*_*i*_ neighbors of *i*. The probability that *i* copies *j* depends on the difference between the payoffs that they obtained in the previous round of the game:

$$\begin{array}{c} 𝓟\left\{x_{j}^{\left(t\right)}\to x_{i}^{\left(t+1\right)}\right\}=\left\{\begin{array}{cc} (\pi_{j}^{\left(t\right)}-\pi_{i}^{\left(t\right)})/\Phi & \text{if}\;\pi_{j}^{\left(t\right)}>\pi_{i}^{\left(t\right)},\\ 0 & \text{otherwise}, \end{array}\right. \end{array}$$ (5)

where Φ is a normalization constant that ensures 𝓟{⋅} ∈ [0, 1]. Note that because of the imitation mechanism of PI, the configurations *x*_*i*_ = 1 ∀*i* and *x*_*i*_ = 0 ∀*i* are absorbing states—the system cannot escape from them.

With BR instead players are fully rational and choose their strategy in order to maximize their payoff, given what their neighbors did in the last round. This means that each player *i*, given $x_{N_{i}}^{\left(t\right)}$, computes the payoffs that she would obtain by choosing action 1 (cooperating) or 0 (defecting) at time *t*, respectively ${\tilde{\pi }}_{C}^{\left(t\right)}$ and ${\tilde{\pi }}_{D}^{\left(t\right)}$. Then

$$\begin{array}{c} x_{i}^{\left(t+1\right)}=\left\{\begin{array}{cc} 1 & \text{if}\;{\tilde{\pi }}_{C}^{\left(t\right)}>{\tilde{\pi }}_{D}^{\left(t\right)},\\ 0 & \text{if}\;{\tilde{\pi }}_{C}^{\left(t\right)}<{\tilde{\pi }}_{D}^{\left(t\right)},\\ x_{i}^{\left(t\right)} & \text{if}\;{\tilde{\pi }}_{C}^{\left(t\right)}={\tilde{\pi }}_{D}^{\left(t\right)}, \end{array}\right. \end{array}$$ (6)

Note that we use PI because it is equivalent, for a well-mixed population, to the well-known replicator dynamics [21], and BR because it is widely used in the economic literature. Note also that whereas a Nash equilibrium is stable by definition under BR dynamics, with PI this is not necessarily true: players can change action by copying better-performing neighbors, even if such a change deteriorates their payoffs. Hence in the latter case a potential stationary state of the system does not necessarily correspond to a Nash equilibrium (unless the network is a complete graph [21]).

### Network Structures

In this study we consider two representative kinds of population structures—with very different degree distribution *P*(*k*): Erdös-Rényi (ER) random graphs [28] and scale-free (SF) random networks [29]. ER graphs are built by adding a link between each pair of nodes independently and with probability *p*. The resulting networks are homogeneous, with *P*(*k*) decaying exponentially for large degrees *k* and the average degree is $\bar{k}=(n-1)p≕m$. SF networks are instead generated using a configuration model [30] with a constraint $k_{max}<\sqrt{n}$, which gives rise to uncorrelated random networks [31] with degree distribution *P*(*k*) ∝ *k* ^−*γ*^. The average degree depends on the network size *n* and converges to a finite value as *n* diverges

$$\begin{array}{c} \bar{k}=\frac{\gamma -1}{\gamma -2}\left[\frac{{\sqrt{n}}^{2-\gamma }-k_{min}^{2-\gamma }}{{\sqrt{n}}^{1-\gamma }-k_{min}^{1-\gamma }}\right]\underset{n\to \infty }{\to }\frac{\gamma -1}{\gamma -2}\;k_{min}≕m. \end{array}$$

### Numerical Simulations

As stated in the introduction, we use a numerical simulation program in order to study our framework of iterated evolutionary games. We start with a fraction *ρ*_0_ of players randomly chosen to undertake action *x* = 1. At each round *t* of the game players collect their payoff *π* ^(*t*)^—given by Equation (1) and Equation (2) respectively for strategic substitutes and complements; then a fraction *q* of players update their strategy—according to either PI or BR. By denoting as *u* ^(*t*)^ the fraction of unsatisfied players (*i.e.*, who would obtain higher payoff by unilaterally switching action) at round *t*, the condition *u* ≡ 0 indicates that the system converged to a Nash equilibrium, defined exactly as a state in which no player can increase her payoff by unilaterally changing her strategy. In these cases, we change by hand the strategy of a random player in order to continue exploring other strategy configurations.

We conclude this section with an overview on simulation parameters. Without loss of generality, we set *ρ*_0_ = 1/2, *c* = 1/2 and *q* = 1/10—but our results remain valid for all values of these parameters in (0, 1). Note that we have to impose the condition *q* < 1 to avoid possible trapping into period-2 loops, where all players change action simultaneously at each round of the game, that can arise in anti-coordination under BR. Concerning the population structure, while we explore various network sizes and link densities for ER graphs, for SF networks we set *γ* = 2.5 and *k*_*min*_ = 3, and consider various sizes: $n=10^{3}\;(\bar{k}=6.415)$, $n=10^{4}\;(\bar{k}=7.48)$, $n=10^{5}\;(\bar{k}=8.13)$, $n=10^{6}\;(\bar{k}=8.51)$—note that for *n* → ∞ it is $m≔{\bar{k}}_{\infty }=9$. All results are averaged over 𝓝=20 independent realizations of the system, an amount that we found enough to tame single-realization fluctuations (that are however shown with error bars, when visible).

## Results

We present now the outcomes of our numerical study, accompanied by a brief summary of the analytical predictions we obtained in [26] as tools to understand the results. In this respect, note that the MF approach (which should work best for homogeneous random graphs) deals with the aggregate density of cooperators *ρ*(*t*), whereas, the HMF (which is more appropriate for heterogeneous networks) involves the computation of the quantity $\Theta (t)=\sum_{k}kP(k)\rho_{k}(t)/\bar{k}$ (*i.e.*, the average of the probability *ρ*_*k*_ that a node of degree *k* cooperates, weighted by the relative degree).

### Best-shot game

#### Proportional Imitation

In this case, the MF prediction is that

$$\begin{array}{c} \rho \left(t\right)={\left[1+\left(\rho_{0}^{-1}-1\right)\exp\left(cqt\right)\right]}^{-1},\;0<\rho_{0}<1. \end{array}$$ (7)

**Fig 1:**
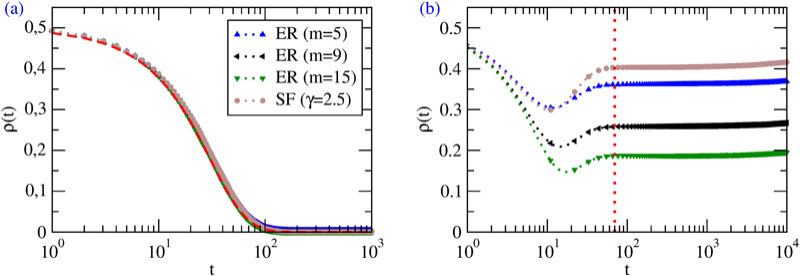
Best-shot games. Average *ρ*(*t*) for ER and SF graphs with *n* = 10^4^ (but results are independent on the specific value of *n*). (a) Results for PI; the red dashed curve is the MF result of Equation (7). (b) Results for BR; the vertical dotted curve approximately divides the dynamics in two regimes: *u*(*t*) > 0 (left) and *u*(*t*) = 0 (right).

Hence the population converges to the state with no cooperators *ρ* = 0 (full defection), unless the initial state is *ρ*_0_ = 1 (full cooperation). We see from Fig. 1a that, also in simulations, the system always goes towards the absorbing state *x*_*i*_ = 0 ∀*i* ⇒ *ρ* ≡ 0 and the full dynamical evolution is in excellent agreement with the MF theory. The convergence toward full defection occurs because a defector cannot copy a neighboring cooperator (who has lower payoff by construction), whereas a cooperator will eventually copy one of her neighboring defectors (who has higher payoff). However, full defection is not a Nash equilibrium as any player surrounded by defectors would do better by cooperating.

Also in the case of SF networks the behavior is remarkably well described by the simple MF approach, which coincides with the HMF as *ρ*_*k*_(*t* = 0) does not depend on *k*.

#### Best Response

For the best-shot game, MF predicts that the final state is, for any initial condition, a mixed state *ρ* = *ρ*_*s*_, where *ρ*_*s*_ ∈ (0, 1) is the solution of the equation:

$$\begin{array}{c} \rho_{s}=e^{-m\rho_{s}}. \end{array}$$ (8)

The HMF approach leads to an analogous conclusion: the final state is Θ_*s*_, which is the solution of the equation

$$\begin{array}{c} \Theta_{s}=\sum_{k}{\left(1-\Theta_{s}\right)}^{k}kP\left(k\right)/\bar{k}. \end{array}$$ (9)

Since BR dynamics leads by construction to Nash equilibria, *ρ*_*s*_ and Θ_*s*_ are the attractors of the dynamics—their values depending only on the average degree of the network (in particular, they decrease for increasing network connectivity) but not on *ρ*_0_, *c* or *q*.

**Fig 2:**
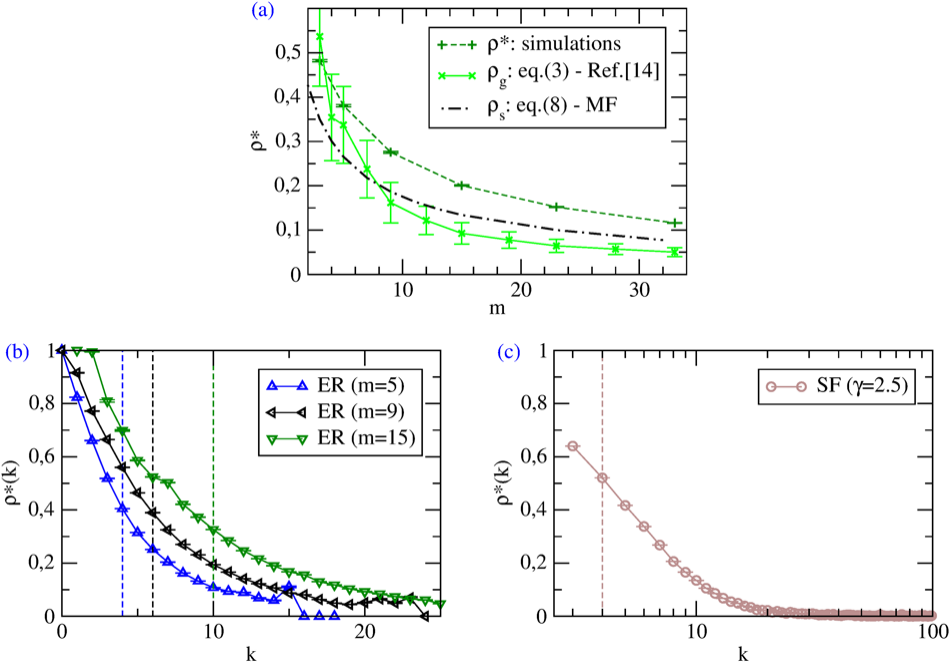
Best-shot games with Best Response. (a) Average ρ* at Nash equilibria vs *m* on ER random graphs, from simulations (*n* = 10^4^), theoretical prediction from Equation (3) and MF Equation (8). (b, c) Average ρ*(*k*) at Nash equilibria for nodes with different degrees *k* (*n* = 10^4^). The vertical dashed lines identify the thresholds *τ* from Equation (3)—which basically depend on *m*.

Numerical simulations confirm such a picture: the dynamics finds a rich variety of Nash equilibria—which are, however, all characterized by an intermediate cooperation level amounting to ρ* (Fig. 1b); moreover, ρ* decreases with increasing network connectivity (Fig. 2a)—in agreement with both Equation (3) and (8). The key observation to understand the features of such equilibria is that, in best-shot games under BR dynamics, a player switches to defection as soon as one of her neighbors is a cooperator, and this happens with higher probability when the player has many social ties (see also Fig. 2b, c). The higher values of ρ* in SF networks than in ER graphs of the same link density (Fig. 1b) can be explained by the presence, in the first case, of more low-degree nodes—who preferentially cooperate as they have few neighbors to exploit. However, low-degree nodes weight less in Θ, so that we find a Θ* for random SF networks similar to the ρ* of ER random graphs. This is in agreement with the mean field predictions: the nodes with highest degrees—that make the difference in *P*(*k*)—do not cooperate in the best-shot game, so that their effects on the system is negligible and *ρ*_*s*_ ≃ Θ_*s*_.

The Nash equilibria found dynamically can be compared with those derived under the assumption of incomplete information by [14]. We first inspect if the cooperation level of our equilibria lies in the range *ρ*_*g*_ ∈ [*ρ*_*k* < *τ*_, *ρ*_*k* ≤ *τ*_], where *ρ*_*k* < *τ*_ and *ρ*_*k* ≤ *τ*_ are the densities of players with *k* < *τ* and *k* ≤ *τ*, respectively, and *τ* is the equilibrium threshold given by Equation (3). Fig. 2a shows the average ρ* at Nash equilibria for ER random graphs as a function of *m*, obtained by numerical simulations, by the theoretical framework of [14], and by the mean field approach. Clearly, whereas the trends are similar, we observe that the cooperation levels of the static equilibria are compatible with simulations only for small values of *m*, while the mean field approximation works better for high average degrees. We then check whether ρ*(*k*), *i.e.*, the strategy profile as a function of the degree *k* of dynamically-found Nash equilibria, is compatible with the analytical strategy profile *σ*(*k*) from Equation (3). Fig. 2b, c shows that, as predicted in [14], ρ*(*k*) is non-increasing; however, the behavior is not step-like. Summing up, the dynamics leads to Nash equilibria which share qualitative features but do not coincide with those identified in [14] under the assumption of incomplete information. Note that this does not occur because the configuration space is not sufficiently explored, but rather because the two approaches are intrinsically different and do not have necessarily to give the same results. In fact, attempts to manually drive our system into one of the equilibria derived by [14] were unsuccessful, and this is because the equilibria that are evolutionary selected by our deterministic BR-based dynamics are indeed proper Nash, whereas, the framework of [14] is probabilistic and selects Bayes-Nash equilibria.

### Coordination game

For coordination games the picture is richer, as the behavior of the system depends on the ratio *α*/*c*. Without loss of generality, we leave *c* = 1/2 fixed and vary the value of *α* in the range (0, *c*).

#### Proportional Imitation

**Fig 3:**
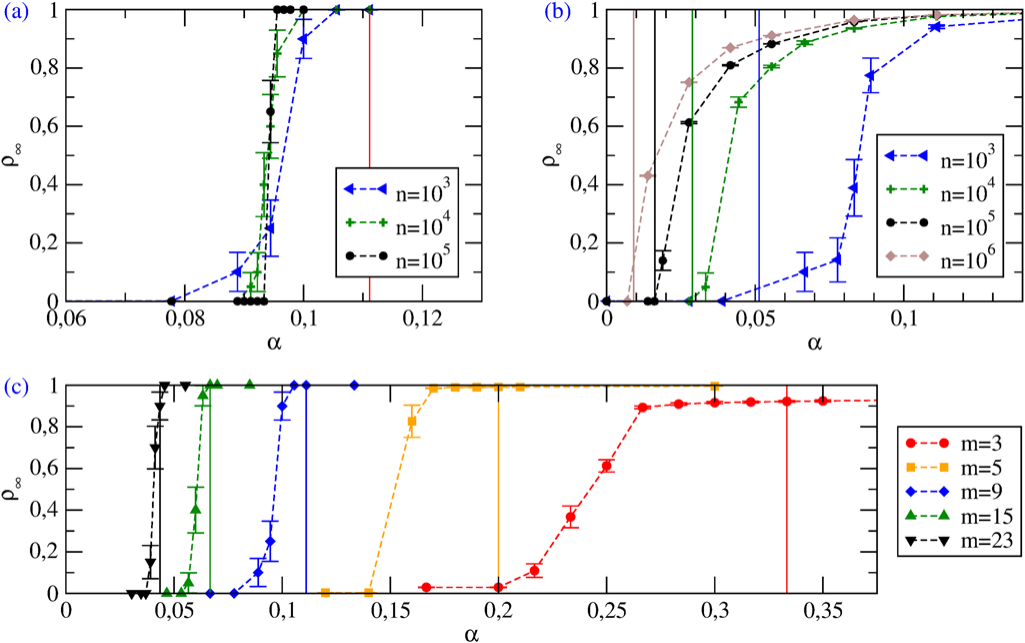
Coordination games with Proportional Imitation. Stationary cooperation levels *ρ*_∞_ vs *α*. The vertical solid lines identify the values of *α*_*c*_. (a) ER graphs with *m* = 9 and various *n*. (b) SF networks with *γ* = 2.5 and various *n*; here *α*_*c*_ values were estimated from Equation (10) as $\alpha_{c}=c/\Theta_{2}≃c\bar{k}/\langle k^{2}\rangle$. (c) ER graphs with *n* = 10^3^ and various *m*. Note that for small values of *m* such that *p* = *m*/(*n*−1) < ln(*n*)/*n* the graph is disconnected: isolated nodes are bound to their initial action, so that full defection and full cooperation are not accessible states; additionally, defective behavior spreads easily inside isolated components which are poorly connected (also for high *α*), so that *ρ*_∞_ remains far from 1.

Here the MF theory predicts the existence of a critical value *α*_*c*_ = *c*/(*mρ*_0_), such that the final state of the dynamics is *ρ* = 0 (full defection) when *α* < *α*_*c*_, and *ρ* = 1 (full cooperation) when *α* > *α*_*c*_. Note, however, that while full defection is always a Nash equilibrium for the coordination game, full cooperation becomes a Nash equilibrium only when *α* > *c*/*k*_*min*_, where *k*_*min*_ is the smallest degree in the network—which means that only networks with *k*_*min*_ > *c*/*α* > 1 may feature a fully cooperative Nash equilibrium. For ER graphs, numerical simulations agree with this picture (Fig. 3a, c), as a transition for *α* = *α*_*T*_ is observed. The value *α*_*T*_ of the transition point found numerically is smaller than the MF prediction *α*_*c*_; however *α*_*T*_ → *α*_*c*_ as the average degree *m* grows. Note that apart from full defection, no other Nash equilibrium (with intermediate cooperation levels) is found.

Also the HMF theory predicts a discontinuous transition between a stable fully defective equilibrium Θ = 0 for *α* < *α*_*c*_ and full cooperation Θ = 1 for *α* > *α*_*c*_ [26]. The critical value is now *α*_*c*_ = *c*/Θ_2_, where

$$\begin{array}{c} \Theta_{2}≔\sum_{k}k^{2}P\left(k\right)\rho_{k}/\bar{k}. \end{array}$$ (10)

The quantity Θ_2_ is related to the second moment of the degree distribution ⟨*k* ^2^⟩ and it can be shown to diverge for networks with *γ* < 3 as the system size *n* goes to infinity. Therefore, at odds with the case of ER random graphs, for SF networks the threshold *α*_*c*_ → 0 as *n* diverges. This vanishing of the transition point is analogous to what occurs for other processes on SF networks, such as percolation or epidemic spreading [32]. This phenomenon is due to the presence of hubs, *i.e.*, nodes with very large degree (diverging with the network size *n*). For any value of *α* > 0, the payoffs *π*_*C*_ of cooperation for the largest hubs will become positive for sufficiently large values of *n*. Those hubs then spread the cooperative strategy to the rest of the network.

Numerical simulations on SF networks confirm only in part the theoretical picture (Fig. 3b). We observe a transition between a fully defective Nash equilibrium, for small values of *α* and final states with intermediate cooperation (which are not Nash equilibria) for *α* > *α*_*T*_. As *α* keeps increasing, cooperation becomes the stable strategy for an increasing number of nodes, and the system heads smoothly towards full cooperation—which in this case becomes a stable Nash equilibrium starting from *α* = *c*/*k*_*min*_ (which is finite as *k*_*min*_ > 0). The transition point *α*_*T*_ tends to zero as the system size grows. Hence HMF theory predicts correctly that the fully defective state disappears in the large size limit (a phenomenon not captured by MF or by the one-shot results in [14]).

#### Best Response

The approximate MF calculations predict again the existence of a critical value *α*_*c*_ = *c*/(*mρ*_0_) such that for *α* ≪ *α*_*c*_ the only Nash equilibrium is full defection, whereas, for *α* ≫ *α*_*c*_ the final state exhibits a large level ρ* of cooperation. Note that here any Nash equilibrium features players with *k* < *c*/*α* being defectors by construction; therefore, the fully cooperative Nash equilibrium is achieved for *α* ≫ *α*_*c*_ but only in networks with *k*_*min*_ > 1.

**Fig 4:**
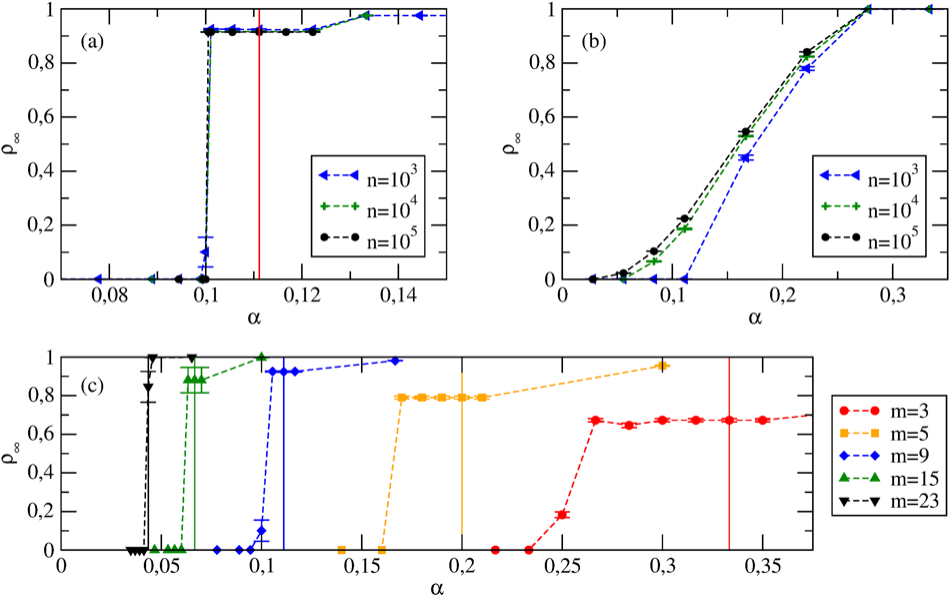
Coordination games with Best Response. Stationary cooperation levels *ρ*_∞_ vs *α*. The vertical solid lines identify the values of *α*_*c*_. (a) ER graphs with *m* = 9 and various *n*. (b) SF networks with *γ* = 2.5 and various *n*. (c) ER graphs with *n* = 10^3^ and various *m*. Note that as *m* decreases, the amount of nodes with *k* < *c*/*α* increases; as these players are defectors by construction, *ρ*_∞_↛ 1 even for *α* → ∞.

The behavior found in numerical simulations is similar to the case of PI (Fig. 4a, c). On ER random graphs a discontinuous transition is found (players have similar degrees, and thus, for a given *ρ*, they become cooperators for similar values of *α*). An important difference is that many non-trivial Nash equilibria (all characterized by intermediate cooperation levels ρ*) are found just above the transition point. A continuous transition is found instead for SF networks (Fig. 4b). Here players have different degrees, and for a given *ρ* each degree class requires its own value of *α* to switch to cooperation.

The HMF approach yields a self-consistent equation for the equilibrium Θ_*s*_:

$$\begin{array}{c} \Theta_{s}=\sum_{k>c/(\alpha \Theta_{s})}kP\left(k\right)/\bar{k} \end{array}$$ (11)

If the network is SF with 2 < *γ* < 3, Θ_*s*_ represents a stable equilibrium whose dependence on *α* is of the form Θ_*s*_ ∼ *α* ^(*γ*−2)/(3−*γ*)^, *i.e.*, there exists a non-vanishing cooperation level Θ_*s*_ no matter how small the value of *α*. However, if the network is more homogeneous (*e.g.*, *γ* > 3), Θ_*s*_ becomes unstable and for *α* → 0 the system always converges to the fully defective Nash equilibria.

**Fig 5:**
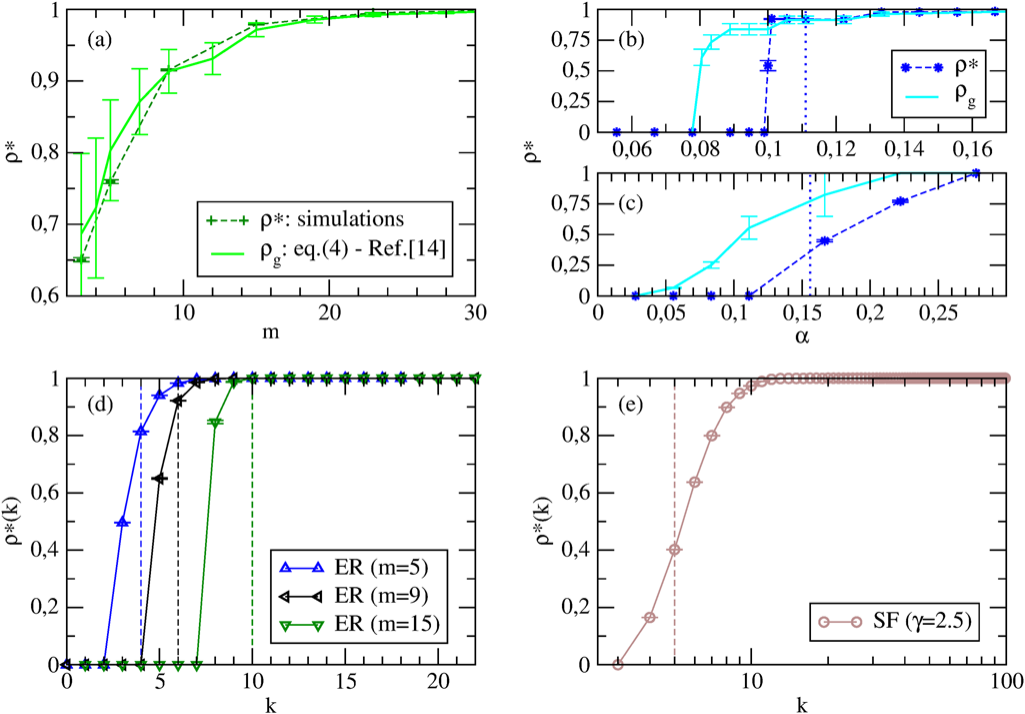
Coordination games with Best Response. Average ρ* at Nash equilibria from simulations and theoretical prediction from Equation (4). (a) ρ* vs *m* for ER random graphs with *n* = 10^4^ at *α* = *α*_*c*_. (b) ρ* vs *α* for ER random graphs with *n* = 10^3^ and *m* = 9. (c) ρ* vs *α* for SF networks with *n* = 10^3^ and *γ* = 2.5. The vertical dashed lines denote the critical value *α*_*c*_. (d, e) Average ρ*(*k*) at *α* = *α*_*c*_ for nodes with different degrees *k* (*n* = 10^4^). The vertical dashed lines identify the thresholds *τ* form Equation (4).

The existence, in some *α* values intervals, of Nash equilibria with intermediate cooperation levels calls for the comparison between simulation results and predictions of [14]. Fig. 5a shows that, for ER graphs at *α* = *α*_*c*_, the cooperation level of equilibria found dynamically lies in the range *ρ*_*g*_ ∈ [*ρ*_*k* ≥ *τ*_, *ρ*_*k* > *τ*_], where *τ* is given by Equation (4). However, we do not find such a good agreement for all values of *α* nor for SF networks (Figs. 5b, c).

Another important prediction of the HMF approach is that *ρ*_*k*_ → 0 when *k* < *c*/(*α*Θ_*s*_), and *ρ*_*k*_ → 1 for *k* > *c*/(*α*Θ_*s*_). In this sense, the equilibria predicted by HMF agree qualitatively with those found in [14]: players’ actions show a non-decreasing dependence on their degrees. Indeed, Fig. 5d, e shows that the average cooperation level ρ*(*k*) of Nash equilibria found dynamically in simulations for *α* = *α*_*c*_ is generally non-decreasing in *k*, and that a step-like behavior of the analytical strategy profile *σ*(*k*) from Equation (4) becomes a good approximation for large *n*. In conclusion, the equilibria predicted in [14] are a good approximation of the equilibria found dynamically only in the thermodynamic limit (*n* → ∞) and, more importantly, only for a small subset of *α* values.

### The effect of correlated networks

In order to study the effects of topologies with degree-degree correlations on the behavior of the games, we run simulations with interaction patterns given by SF networks with assortative and disassortative correlations. To generate these networks, we use the prescriptions of the model by Weber and Porto [33] (again with the constraint $k_{max}<\sqrt{n}$ on the largest degree). Correlated networks feature a parameter *β* that controls the properties of the resulting topologies: the average degree of the nearest-neighbors of a node of degree *k* is proportional to *k* ^*β*^, so that for *β* > 0 neighbors of nodes with large *k* have large degree (assortative networks), while for *β* < 0 nodes with large *k* have neighbors with small degree and vice versa (disassortative networks). The uncorrelated case is recovered for *β* = 0.

**Fig 6:**
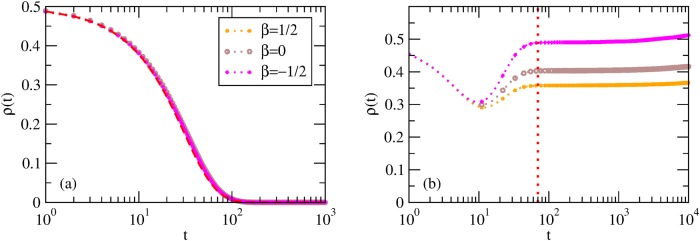
Best-shot games for correlated networks. Here *γ* = 2.5, *m* = 9, *n* = 10^4^ and various *β* (again, these results are independent on the specific value of *n*). (a) *ρ*(*t*) for PI—the red dashed curve being the MF Equation (7). (b) *ρ*(*t*) for BR—the vertical dotted line approximately dividing the dynamics in the regimes *u*(*t*) > 0 (left) and *u*(*t*) = 0 (right).

For the best-shot game (Fig. 6a), the simple case of PI dynamics is totally unaffected by the presence of correlations: the temporal evolution and the asymptotic state do not depend at all on *β*. Instead, when strategies evolve according to BR (Fig. 6b), the dynamics leads to Nash equilibria with levels ρ* of cooperation (dominated by the behavior of low-degree nodes) that decrease with *β*. The reason is that for *β* < 0 low-degree nodes are connected to hubs, which are likely to be defectors; as a consequence they tend to cooperate and ρ* is higher. The opposite occurs in assortative networks (*β* > 0): low-degree nodes are connected to each other and less cooperation is needed.

**Fig 7:**
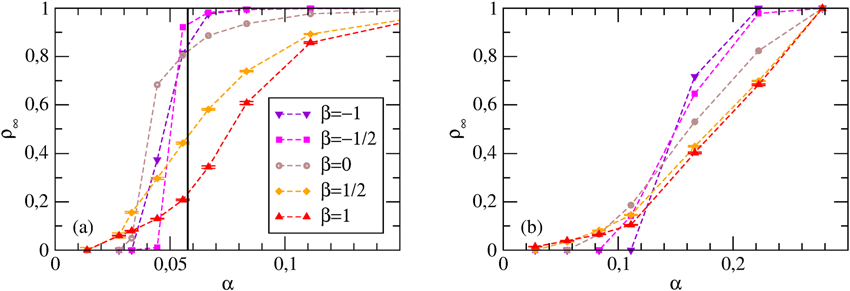
Coordination games for correlated networks. Here with *γ* = 2.5, *m* = 9, *n* = 10^4^ and various *β* (again, these results are independent on the specific value of *n*): Stationary cooperation levels *ρ*_∞_ vs *α* for PI (a) and BR (b) dynamics. The vertical solid line identifies the value of *α*_*c*_ = *c*/Θ_2_(0).

For coordination games with PI dynamics, the effect of assortative (disassortative) correlations is to make broader (sharper) the transition observed as a function of *α* for finite network size *n* (Fig. 7a). This can be understood by considering that, in the assortative case, hubs tend to be connected with each other, so that cooperation can spread more easily among them and be sustained by mutual connections: the critical density of stable cooperators decreases. At the same time, low-degree nodes are connected only with each other, so that they require larger values of *α* to sustain cooperation within themselves (as compared to the uncorrelated case). As a result, the transition becomes broader. An analogous argument explains the sharper transition for dissortative networks. In the case of BR dynamics, the same picture applies (Fig. 7b).

## Discussion

In this work we have studied by numerical simulations two kinds of games, namely the best-shot and the coordination games, as representatives of two wide classes of social interactions (strategic substitutes and strategic complements, respectively). In these games, the welfare of a player depends both on her own actions and on the actions taken by her partners; thus, we have considered different topological structures for the pattern of interactions. We have embedded these games into an evolutionary framework, described by two types of dynamics, widely employed to model evolving populations: PI, in which players imitate more successful neighbors, and BR, in which players are rational and make optimal choices. We have performed numerical simulations, determining the attractors of the evolution, and characterizing them in term of Nash equilibria. By comparison with the results of numerical simulations we have assessed the validity of the MF approaches in describing such systems, gaining additional insight on individual behaviors, and we have also compared our findings with the theoretical predictions of [14] about the features of Nash equilibria in one-shot games under incomplete information.

Generally, we observe that the behavior of the system is highly influenced by the dynamics employed and by the population structure. Strategic substitutes under PI dynamics represents the simplest case, in which full defection is the only accessible (but non-Nash) equilibrium, whatever the underlying topology, in complete agreement with the mean field predictions. This suggests that the failure to find a Nash equilibrium arises from the (bounded rational) dynamics and, with the benefit of hindsight, it is clear that imitation is not a good procedure for players to decide in anti-coordination games. Such a conclusion is supported by the behavior observed for strategic substitutes under BR dynamics. Indeed, here we observe many stable Nash equilibria, with cooperation level ρ* slightly smaller than (but close to) the mean field prediction *ρ*_*c*_. Moreover, in simulations we observe precisely what is predicted by the MF theory, namely that ρ* decreases with increasing network connectivity and does not depend on the initial conditions, game and simulation parameters, and system size (which was taken as infinite in the analytical calculations). Concerning the topology, ρ* is enhanced in heterogeneous networks because of more low-degree nodes who are typically cooperators. Additionally, disassortativity has a positive effect on the cooperation levels, as low-degree nodes are connected to high-degree nodes who are likely to be defectors, and thus tend to cooperate even more. Vice versa, assortativity allows more low-degree nodes to defect.

The picture is far more rich and interesting for strategic complements, that feature an additional parameter *α* playing a key role in determining which equilibria are dynamically accessible. Indeed, in the case of PI for ER random graphs we observe two kinds of stationary states: fully defective Nash equilibria for *α* < *α*_*T*_, and full cooperation for *α* > *α*_*T*_ (that becomes Nash equilibrium only when *α* > *c*/*k*_*min*_). Remarkably, *α*_*T*_ → *α*_*c*_ for *n*, *m* → ∞, where *α*_*c*_ = *c*/(*mρ*_0_) is the value predicted by the MF theory. We thus see that imitation is indeed a good procedure to choose actions in a coordination setup: PI does lead to Nash equilibria, and indeed it makes a very precise prediction: a unique equilibrium that depends on the initial density. If the topology is a SF network, HMF theory and simulations agree on *α*_*T*_ and *α*_*c*_ going to zero for *n* → ∞, indicating that in these cases cooperation emerges also when the incentive to cooperate vanishes. The situation with BR dynamics is rather similar to the case of PI, with the single difference that for ER random graphs and *α* > *α*_*c*_ the stationary state is now a Nash equilibrium, and again full cooperation is achieved for *α* > *c*/*k*_*min*_. Thus we see that, with BR, equilibria with intermediate values of the density of cooperators are obtained in a range of initial densities. Compared to the situation with PI, in which we only find the absorbing states as equilibria, this points to the fact that more rational players can eventually converge to equilibria with higher payoffs. Finally, we have found that, whatever the dynamics, the presence of topological correlations in the network does not change the global qualitative picture.

Besides the general features of the dynamics, we have also been able to study the degree-dependent features of Nash equilibria (when present) and compare our findings with the theoretical predictions of [14], in the which authors consider games played once and for all but using only partial information on the underlying topology. Our conclusion is that, while the Nash equilibria predicted in [14] cannot (always) be reached (*i.e.*, such equilibria, arising in a one-shot game, are not necessarily the outcome of an evolutionary process in the iterated version of the game), their theory still provides good guidance on the shape and features of the Nash equilibria which are evolutionarily accessible. Note that the connection between the two visions of the game is not trivial, and even less so in our networked context. We refer the reader to [20, 21] for a discussion on dynamical approaches to Nash equilibria from a rigorous viewpoint.
